# Molecular evolution and the global reemergence of enterovirus D68 by genome-wide analysis

**DOI:** 10.1097/MD.0000000000004416

**Published:** 2016-08-07

**Authors:** Yu-Nong Gong, Shu-Li Yang, Shin-Ru Shih, Yhu-Chering Huang, Pi-Yueh Chang, Chung-Guei Huang, Kuo-Chin Kao, Han-Chung Hu, Yi-Chun Liu, Kuo-Chien Tsao

**Affiliations:** a Department of Laboratory Medicine, Linkou Chang Gung Memorial Hospital; b Department of Medical Biotechnology and Laboratory Science; c Research Center for Emerging Viral Infections, Chang Gung University; d Department of Pediatrics, Linkou Chang Gung Memorial Hospital; e College of Medicine, Chang Gung University; f Department of Respiratory Therapy; g Department of Pulmonary and Critical Care Medicine, Linkou Chang Gung Memorial Hospital; h Department of Respiratory Therapy; i Department of Pulmonary and Critical Care Medicine, Chang Gung University, Taoyuan, Taiwan.

**Keywords:** enterovirus D68, genetic diversity, global reemergence, molecular evolution, phylogenetic tree, whole genome

## Abstract

Human enterovirus D68 (EV-D68) was first reported in the United States in 1962; thereafter, a few cases were reported from 1970 to 2005, but 2 outbreaks occurred in the Philippines (2008) and the United States (2014). However, little is known regarding the molecular evolution of this globally reemerging virus due to a lack of whole-genome sequences and analyses. Here, all publically available sequences including 147 full and 1248 partial genomes from GenBank were collected and compared at the clade and subclade level; 11 whole genomes isolated in Taiwan (TW) in 2014 were also added to the database. Phylogenetic trees were constructed to identify a new subclade, B3, and represent clade circulations among strains. Nucleotide sequence identities of the *VP1* gene were 94% to 95% based on a comparison of subclade B3 to B1 and B2 and 87% to 91% when comparing A, C, and D. The patterns of clade circulation need to be clarified to improve global monitoring of EV-D68, even though this virus showed lower diversity among clades compared with the common enterovirus EV-71. Notably, severe cases isolated from Taiwan and China in 2014 were found in subclade B3. One severe case from Taiwan occurred in a female patient with underlying angioimmunoblastic T-cell lymphoma, from whom a bronchoalveolar lavage specimen was obtained. Although host factors play a key role in disease severity, we cannot exclude the possibility that EV-D68 may trigger clinical symptoms or death. To further investigate the genetic diversity of EV-D68, we reported 34 amino acid (aa) polymorphisms identified by comparing subclade B3 to B1 and B2. Clade D strains had a 1-aa deletion and a 2-aa insertion in the *VP1* gene, and 1 of our TW/2014 strains had a shorter deletion in the 5′ untranslated region than a previously reported deletion. In summary, a new subclade, genetic indels, and polymorphisms in global strains were discovered elucidating evolutionary and epidemiological trends of EV-D68, and 11 genomes were added to the database. Virus variants may contribute to disease severity and clinical manifestations, and further studies are needed to investigate the associations between genetic diversity and clinical outcomes.

## 1 Introduction

Human enterovirus D68 (EV-D68) belongs to the family Picornaviridae and the genus *Enterovirus*, which was first isolated in California in 1962.^[1]^ After the first case, 26 EV-D68 cases were sporadically reported from 1970 to 2005.^[2]^ However, from October 2008 to March 2009, an outbreak was detected in the Philippines among pediatric patients hospitalized with pneumonia.^[3]^ Since then, this virus has been recognized as a reemerging pathogen.^[4]^ Studies indicated an increasing number of EV-D68-positive cases associated with acute respiratory illness in Asia, Europe, and the United States; some cases were also associated with central nervous system diseases.^[5–9]^ Notably, the largest outbreak in the United States caused 130 laboratory-confirmed cases in September 2014.^[10]^

The enterovirus genome consists of approximately 7500 nucleotides (nt) encoding a polyprotein, from which 11 viral genes are translated with the characteristic gene order 5′-*VP4*, *VP2*, *VP3*, *VP1*, *2A*, *2B*, *2C*, *3A*, *3B*, *3C*^*pro*^, and *3D*^*pol*^-3′. Among them, the *VP1* gene has been used to distinguish various enterovirus serotypes,^[11–13]^ and phylogenetic analysis has been used to discriminate lineages and detect new or emerging strains, including recently reported subclades B1 and B2 and clade D.^[14–16]^ It suggested that interclade variations led to the identification of new clade, which in *VP1* gene may alter viral antigenicity.^[16]^ The *VP1* gene contains serotype-specific neutralization sites (e.g., the BC loop), which are located at the carboxyl end of the protein and associated with viral antigenicity.^[5]^ Although 1 *VP1* deletion in clade-A strains^[5]^ and 1 *VP1* insertion in the strain 1737-Yamagata-2008^[17]^ have been reported, further studies are required to explore the association between genetic characteristics and disease severity. In addition to the *VP1* gene, EV-D68 genomes from the early 1960s to mid-1990s underwent a rearrangement in the spacer region of the 5′ untranslated region (UTR) between the end of the internal ribosome entry site and the polyprotein open reading frame (ORF).^[5]^ The rearrangement resulted in 2 deletions of 24 and 11 nt in the spacer region, which might have a significant effect on the initiation of translation. Although the virulence was affected by the variations within the internal ribosome entry site,^[18,19]^ the role of the spacer region with respect to viral fitness is not well known. In brief, genetic mutations may affect virulence by enhancing translational efficiency and correlate with the recent increase in EV-D68 cases worldwide.

Enteroviruses (e.g., EV-71) in Taiwan (TW) commonly circulate in the summer; however, an immunofluorescence assay for EV-D68 is not available, and little is known about the molecular genetics and epidemiology of EV-D68 strains in Taiwan. A previous study provided the sequences of 29 *VP1* genes from EV-D68/TW from 2007 to 2014.^[20]^ The authors indicated that EV-D68 has been endemic in Taiwan. Because they included only *VP1* sequences, further studies were required to understand the genetic characteristics of whole genomes and the association between EV-D68 and severe clinical disease.

The primary goal of the current study was to investigate the molecular phylogeny, diversity, and epidemiology of EV-D68 strains from around the world. To this aim, we performed phylogenetic and genetic diversity analyses on all sequences available from GenBank as well as 11 EV-D68/TW strains isolated in 2014, which were sequenced for this study. Sequences were compared at the clade and subclade level.

## 2 Methods

### 2.1 Ethics statement

This study was approved by the Institutional Review Board of Chang Gung Medical Foundation, Linkou Medical Center, Taoyuan, Taiwan, with approval number 104-2536B.

### 2.2 Viral RNA isolation and PCR amplification for sequencing EV-D68 genomes

Eleven viral isolates were collected in Taiwan in 2014 for this study, and a further 136 complete/near-complete and 1248 partial genomes of EV-D68 were retrieved from GenBank in December 2015 for analysis. The 11 isolates include 3 from Linkou Chang Gung Memorial Hospital (CGMH), a medical center in northern Taiwan that processed a considerable number of clinical specimens for virus surveillance every year. The other 8 were from the The Taiwan Centers for Disease Control (Taiwan CDC), to which clinical virus isolates from 11 nationwide contracted laboratories for virus surveillance in Taiwan were sent. According to the manufacturer's specifications, RNA was extracted using the LabTurbo RNA Extraction Kit (TaiGen Biotechnology Inc, Taipei, Taiwan). cDNA was synthesized from total RNA using the SuperScript III reverse-transcription system (Invitrogen Corp, Carlsbad, CA). RNA was amplified and sequenced using 6 primer sets, as shown in Table 1. The polymerase chain reaction (PCR) mixture contained 5 μL of cDNA, 0.5 μM of each primer, and KOD plus Taq DNA polymerase (TOYOBO, Osaka, Japan) and was adjusted to a final volume of 25 μL with nuclease-free water. Each round of PCR amplification was performed under the following conditions: an initial denaturation at 94°C for 3 minutes was followed by 35 amplification cycles consisting of 94°C for 30 seconds, 55°C for 30 seconds, and 72°C for 3 minutes and concluded with a final extension at 72°C for 7 minutes. According to the manufacturer's specifications, PCR products were purified from agarose gel using a gel extraction kit (QIAGEN, Hilden, Germany). Purified DNA served as the template for the chain termination reaction with the ABI 3730 XL DNA Analyzer (Applied Biosystems Inc, Foster City, CA). The 11 EV-D68 genomes sequenced in this study have been deposited in GenBank with accession numbers KT711078 to KT711088. Table 2 shows the accession numbers and strain names and also presents clinical and demographic data for future investigations.

**Table 1:**
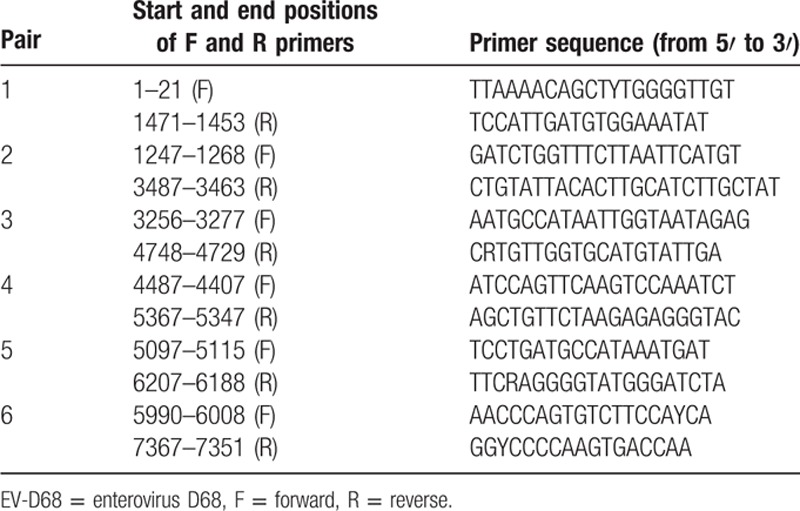
Six primer pairs for amplifying and sequencing EV-D68 genomes.

**Table 2:**
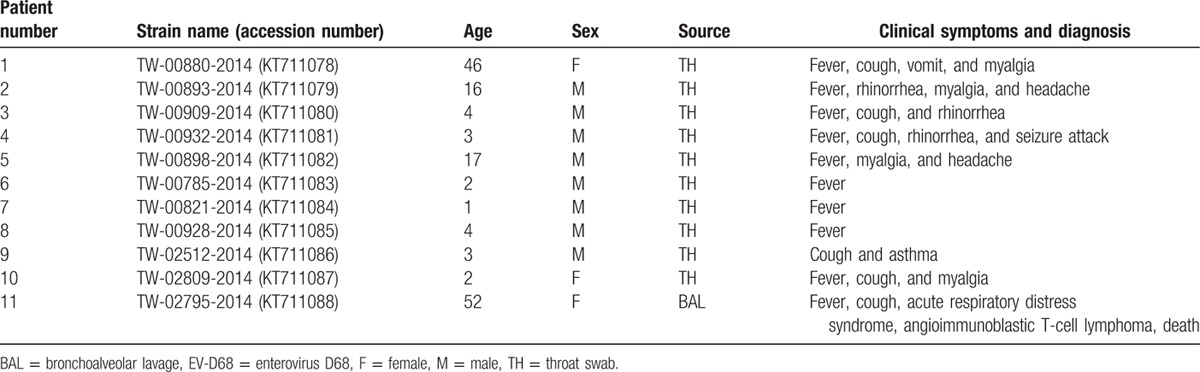
Clinical and demographic symptoms of 11 EV-D68 infections.

### 2.3 Phylogenetic and sequence analysis of EV-D68 genomes

A total of 147 full and 1248 partial sequences of EV-D68 were collected for analysis. These sequences were aligned using Clustal Omega version 1.2^[21]^ to explore indels in the *VP1* gene, with special attention paid to those in Taiwan. To investigate the molecular phylogeny of strains circulating globally, phylogenetic trees were generated using Bayesian Evolutionary Analysis Sampling Trees 2 package^[22]^ on *VP1* sequences from strains collected in Taiwan, the United States, China, Japan, Mexico, Canada, France, New Zealand, and Haiti. A maximum clade credibility (MCC) tree under the Hasegawa–Kishino–Yano model was estimated. Thirty million Markov chain Monte Carlo chains with 10% burn-in were generated. One MCC tree was constructed for every 3000 chains, and a single consensus tree was summarized from these MCC trees. In addition, all position-specific polymorphisms and substitutions identified by sequence analysis were plotted on graphs using WebLogo 3.^[23]^

## 3 Results

### 3.1 Clinical and demographic data from EV-D68–positive cases

Table 2 summarizes the demographics and clinical symptoms of the 11 patients, including 8 males and 3 females. Nine patients were younger than 18 years of age, and the other 2 were 46 and 52 years old. Ten specimens were derived from throat swabs, and 1 was from a bronchoalveolar lavage (BAL). Most of the patients presented with a respiratory infection, and common signs and symptoms included fever (10/11, 91%), cough (6/11, 55%), myalgia (4/11, 36%), rhinorrhea (3/11, 27%), vomiting (1/11, 9%), and headache (1/11, 9%). Case 4, a 3-year-old boy, had a seizure. Case 11, a 52-year-old female, had underlying angioimmunoblastic T-cell lymphoma (AITL) diagnosed in March 2014 and subsequently received chemotherapy in partial response to her status. She was admitted to the hospital due to disseminated herpes zoster over the skin in late August. After admission, she developed bilateral pneumonia with respiratory failure; this disease was accompanied by moderate acute respiratory distress syndrome (ARDS) with a PaO_2_/FiO_2_ ratio of 130 mm Hg and 8 cm H_2_O positive end-expiratory pressure on September 8. She received a bronchoscopy with BAL for the pneumonia diagnostic workup the next day. There were no bacteria, fungi, *Pneumocystis jirovecii*, *Mycobacterium tuberculosis*, influenza virus, herpes simplex virus, or cytomegalovirus, but EV-D68 was identified. Despite vigorous supportive care, her condition deteriorated, and she eventually died on October 10. Although there was no direct evidence of EV-D68 causing this death, we could not exclude the possibility that EV-D68 may have triggered clinical symptoms or death.

### 3.2 Phylogenetic analysis of the *VP1* gene

**Figure 1:**
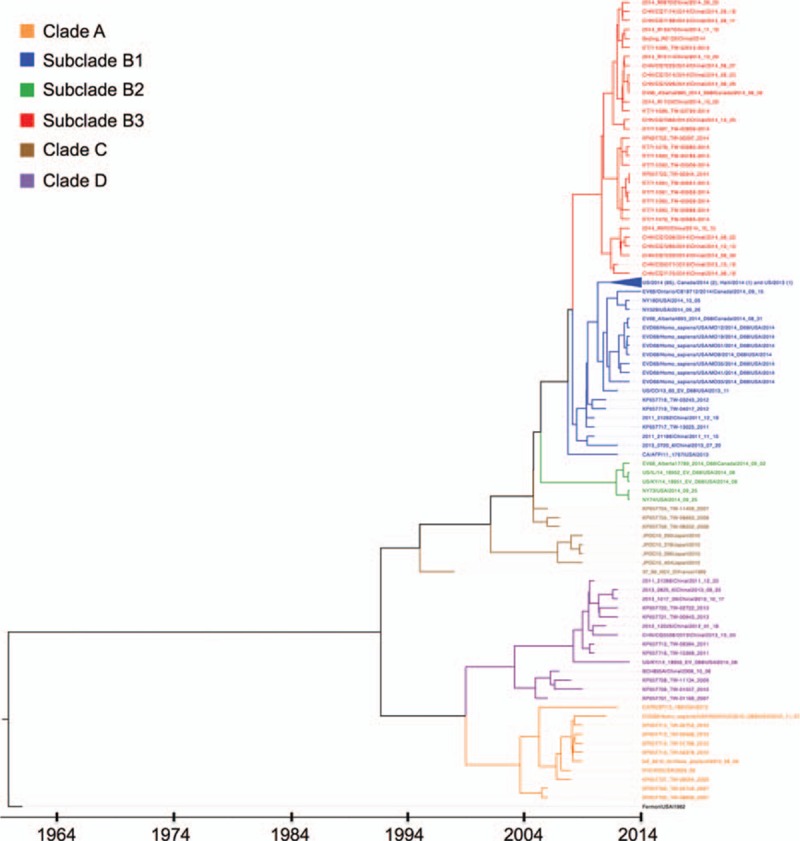
A maximum clade credibility tree by Bayesian phylogenetic analysis. A total of 169 EV-D68 strains were collected, including 147 complete and nearly complete genomes (including 11 TW strains from this study) and 22 TW strains in partial length, from 2007 to 2014. This phylogenetic tree based on the *VP1* gene (933 nt long) presented 4 major clades, A to D. Clade B was further divided into B1 to B3. All TW/2014 and China/2014 strains were found in a new subclade, B3. The node label includes the name, country, and year of strains separated by vertical bars. The colors of the node and branch labels depend on their clades. A total of 84 US/2014, 4 Canada/2014, 1 Haiti/2014, and 1 US/2013 strains in subclade B1 are represented by a triangle; their sequence counts are shown in parentheses. EV-D68 = enterovirus D68.

We collected a total of 169 EV-D68 strains, including 147 complete/nearly complete and 22 partial TW sequences from 2007 to 2014. A phylogenetic tree was generated based on their *VP1* gene, which is 933 nt long, as shown in Fig. 1. All 13 TW/2014 strains, including 2 severe cases, were classified in a new subclade, B3, grouping with the 1 Canada/2014 and 17 China/2014 strains. Among the Chinese cases, 7 patients (accession numbers as KP240936, KT803598, KT803592, KT803594, KT803599, KT803596, and KT803600) had moderate or severe asthma and pneumonia.^[24,25]^ The results also demonstrated that various clades of this reemerging pathogen were circulating in Taiwan, and cocirculated within a given year. For example, TW strains in clade D were isolated in 2007, 2009, 2010, 2011, and 2013, and TW/2007 strains clustered in clades A, C, and D. In addition to TW strains, clade B1 contains 97 US/2014/2013, 4 Canada/2014, 3 China/2011/2013, 2 Mexico/2014, and 1 Haiti/2014 strains; clade A contains 3 US/2009/2012/2013 strains and 1 New Zealand strain; clade C contains 4 Japan/2010 strains and 1 France strain; and clade D contains 6 China/2008/2011/2012/2013/2014 strains and 1 US/2014 strain. An intraclade recombination was detected in strain US.KY.14.18951,^[15]^ which grouped into subclade B2, clustering with 3 US/2014 strains and 1 Canada/2014 strain.

### 3.3 Sequence identities among clades and subclades

To compare subclade B3 strains to A, B1, B2, C, and D strains, we collected 129 complete genomes to calculate their nucleotide identities based on each segment and ORF. Table 3 shows average nucleotide identities of 97.7% to 98.6% in the P1 region (from *VP4* to *VP1*), 98.2% to 98.9% in P2 (from *2A* to *2C*), and 98.3% to 99.2% in P3 (from *3A* to *3D*) within subclade B3. Comparing B3 to B1 and B2, average nucleotide identities of 93.0% to 95.9%, 93.4% to 96.0%, and 91.2% to 97.7% in the P1, P2, and P3 regions were observed, respectively. Comparing B3 to A, average nucleotide identities of 87.9% to 90.0%, 91.0% to 92.5%, and 87.2% to 89.7% were found in the P1, P2, and P3 regions, respectively. Similar identities were observed in the comparison of B3 to C and D.

**Table 3:**
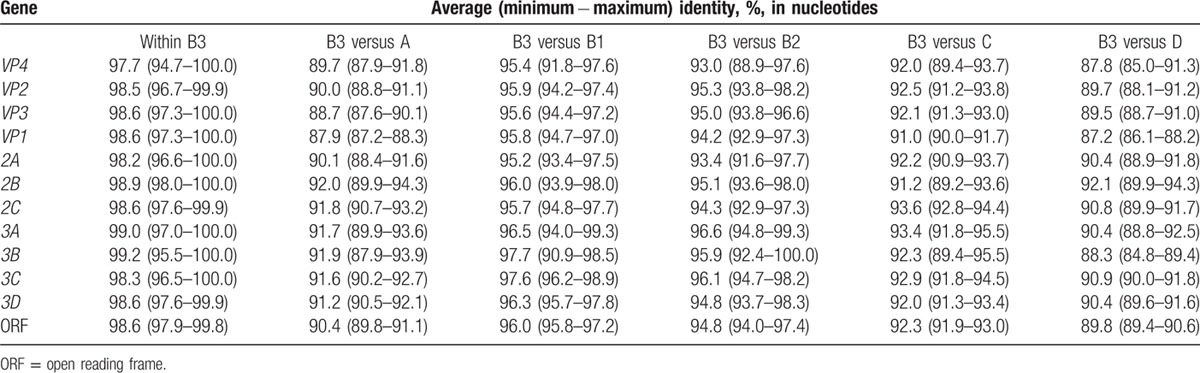
Sequence identities based on each gene and ORF comparing subclade B3 with other clades.

### 3.4 Genetic diversity of EV-D68 genomes

**Figure 2:**
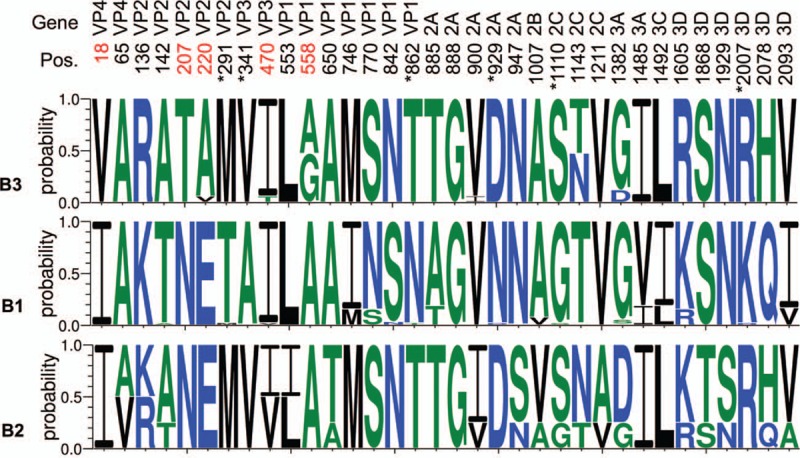
The 34 amino acid polymorphisms of clade B. Thirty-four amino acid polymorphisms were identified from EV-D68 genomes in a length of approximately 2190 aa, including 7 in *VP1*, 6 in *3D*, 5 in *2A*, 5 in *VP2*, 3 in *3C*, 2 each in *VP4*, *VP3*, and *3A*, and 1 each in *2B* and *3C*. Five positions (i.e., V18, T207, A/V220, T470, and G558) were newly reported by comparing subclade B3 with B1 and B2; these positions are marked in red. A previous study reported 6 polymorphisms (i.e., M291, V341, T860, D927, S1108, and R2005 in subclade B3), which are marked with a superscript asterisk. Aa = amino acid, EV-D68 = enterovirus D68.

To further characterize the newly reported subclade B3, we compared the consensus sequence of B3 to B1 and B2 based on 129 complete genomes and identified 34 amino acid substitutions, including 7 in *VP1*, 6 in *3D*, 5 in *2A*, 5 in *VP2*, 3 in *3C*, 2 each in *VP4*, *VP3*, and *3A*, and 1 each in *2B* and *3C*, as shown in Fig. 2. Five positions in the B3 subclade (i.e., V18, T207, A/V220, T470, and G558) involved novel substitutions compared with subclades B1 and B2. Six polymorphisms (i.e., positions at 291, 341, 860, 927, 1108, and 2005) have been reported^[14]^ that in subclade B3 were identical to those in subclade B2, although position 1108 presented as both S and G in subclade B2.

**Figure 3:**
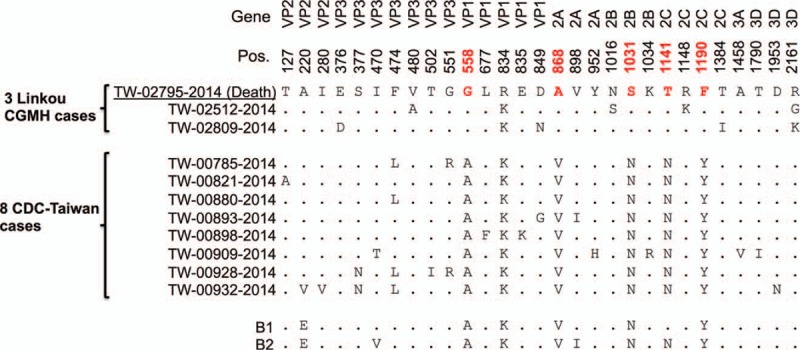
The 29 nonconserved residues of 11 Taiwan/2014 genomes. Twenty-nine nonconserved residues were identified, including 7 in *VP3*, 5 in *VP1*, 4 in *2C*, 3 each in *VP2*, *2A*, *2B*, and *3D*, and 1 in *3A*. These residues in the same aligned column were identical to the first sequence TW-02795-2014 and are masked by dots. Position 474 was the most divergent site, with 7 F's and 4 L's, followed by positions 558, 868, 1031, 1141, and 1190 with 3 substitutions, positions 377, 551, 849, and 2161 with 2 substitutions, and another 19 sites with only 1 substitution. Positions 558, 868, 1031, 1141, and 1190 are marked in red; they exhibit residues “G,” “A,” “S,” “T,” and “F” in 3 Linkou CGMH cases, but “A,” “V,” “N,” “N,” and “Y” in the other 8 TW cases and subclades B1 and B2, except for position 1141 in subclade B1. CDC = Centers for Disease Control and Prevention, CGMH = Chang Gung Memorial Hospital.

As described in Table 3, clade B strains showed high sequence identities. Figure 3 presents 29 nonconserved residues from the 11 complete genomes provided in this study. Among them, position 474 had the highest diversity with 7 F's and 4 L's, followed by 5 positions (i.e., 558, 868, 1031, 1141, and 1190) with 3 substitutions, 4 positions (i.e., 377, 551, 849, and 2161) with 2 substitutions, and another 19 sites with only 1 substitution. Furthermore, positions 558, 868, 1031, 1141, and 1190 in 3 Linkou CGMH cases had residues “G,” “A,” “S,” “T,” and “F,” respectively, which diverged from the other 8 TW strains that had residues “A,” “V,” “N,” “N,” and “Y.” One death occurred among the 3 Linkou CGMH patients, associated with underlying AITL.

An “N” residue deletion at position 692 and “RL” residue insertion at positions 860 and 861 in *VP1* have been reported.^[6,17]^ We further explored the existence of genetic indels based on the phylogenetic clades. Notably, 5 China 2008/2011/2013/2014 strains and 1 US/2014 strain in clade D have an N692 deletion and R860/L861 insertions in *VP1*. Because 7 TW strains in clade D have incomplete *VP1* sequences, we could not confirm the insertion event, but they all have an N692 deletion. In clade A, 7 TW 2007/2009/2010 strains, 3 US 2009/2012/2013 strains, and 1 New Zealand strain have an N692 deletion. Clades B and C did not have deletions. To conclude, clade D has an N692 deletion and R860/L861 insertions; clade A has an N692 deletion, and clades B and C have neither. In addition to those sequences, which were associated with specific clades, we discovered 5 France strains (CF254007, CF253080, CF307029, CF298012, and CF289007), 1 Italy strain (ITA/23341/14), and 1 Japan strain (1737-Yamagata-2008) that have an N692 deletion and R860/L861 insertions.

Two deletions of 24 and 11 nucleotides in the 5′ UTR have been reported.^[7]^ One deletion was located from positions 681 to 704 and was observed in all 11 TW/2014 strains in this study. The other deletion was located from positions 721 to 731 in the 5′ UTR and was observed in all TW/2014 strains except for TW-00880-2014. This strain has only an 8-nt deletion, not the previously reported 11-nt one.

## 4 Discussion

Although EV-D68 was first described in the United States in 1962 and few cases were reported until 2005, sporadic outbreaks were detected in the United States, the Netherlands, the Philippines, and Japan from 2008 to 2010.^[1–3,6–8]^ In 2014, the largest outbreak to date of this reemerging virus in the United States and Canada was reported, and subsequent studies described respiratory diseases in Taiwan, China, the Philippines, and Europe.^[10,24–27]^ However, understanding of this virus remains limited due to a lack of whole genome and molecular evolution analyses of strains from around the globe. This study involved a genome-wide analysis of globally circulating strains with sequences downloaded from GenBank and contributed 11 EV-D68/2014 genomes isolated in Taiwan, including 2 severe cases (i.e., cases 4 and 11 in Table 2). A total of 169 strains from around the world were used to generate a tree that included a new subclade, B3, along with previously reported clades A, B1, B2, C, and D^[6,14–16]^ (Fig. 1). All TW/2014 and China/2014 strains belong to subclade B3, and several cases in China were associated with moderate or severe asthma and pneumonia.^[24,25]^Figure 1 also demonstrates that various clades of this reemerging pathogen cocirculated in Taiwan; for example, TW strains in clade D were isolated in 2007, 2009, 2010, 2011, and 2013, and TW/2007 strains clustered in clades A, C, and D. To further compare clades and subclades, we calculated sequence identities based on each gene and ORF using all the complete genomes. Based on the *VP1* gene, average sequence identities of 95.8% and 94.2% were observed by comparing subclade B3 to B1 and B2, respectively, and sequence identities of 87.9%, 91.0%, and 87.2% were found by comparing B3 to A, C, and D, respectively. Similarly, genotype replacements of EV-71 (one of the most common enteroviruses) in Taiwan, Vietnam, and Malaysia have been reported; for example, the predominant genotypes were C2 in 1998, B4 in 1999 to 2003, C4a in 2004 to 2005, 2008, and 2012, and B5 in 2008 to 2009 and 2011 in Taiwan.^[28–30]^ An average sequence identity of 88.7% based on the *VP1* gene was observed by comparing subgenotype C5 to subclades from C1 and C4, and 82.1% by comparing to genotypes A and B1 to B5.^[31]^ Although the sequence diversity of the *VP1* gene among EV-D68 clades was lower than that among EV-71 genotypes, the phenomena of genotype and clade replacement need to be clarified to better monitor the reemergence of EV-D68, as evidenced by previous outbreaks.

In this study, case 11, with underlying AITL, developed ARDS and eventually died. The BAL specimen collected to test for pneumonia revealed no other possible pathogen; only EV-D68 was identified. Regarding the cause of ARDS, EV-D68 associated with ARDS in adults is very rare, and only 1 case was reported in the United States in 2014.^[32]^ In a seroepidemiological study in 2010 in Finland,^[33]^ Smura et al suggested that after EV-D68 infection, most adults generate neutralizing antibodies; however, host factors (e.g., underlying disease) play a key role in determining disease severity. At the other end of the age spectrum, 7 of 11 TW cases occurred in children younger than 5 years of age. Case 4, the other severe case, occurred in a child who was 3 years old and had a seizure. One study reported a geographically and temporally defined cluster of acute flaccid paralysis and cranial nerve dysfunction in children associated with an EV-D68 outbreak with respiratory illness.^[34]^ The authors suggested the possibility of an association between EV-D68 and neurological disease in children. These observations indicated that infants, children, and teenagers have a higher risk of EV-D68 infection than adults.

A previous study focused on investigating the association between EV-D68 infections and acute flaccid myelitis.^[14]^ The authors reported 6 polymorphisms (T291, A341, N860, N927, G1108, and K2005) identified from EV-D68 US outbreak strains; of these 5 were also involved in neuropathogenic poliovirus, enterovirus D70, or both. These 6 polymorphisms, M291, V341, T860, D927, S1108, and R2005 in Taiwanese strains, diverged from those US/2014 strains (i.e., from patients with acute flaccid myelitis), as shown in Fig. 2. Although these 6 polymorphisms in TW/2014 and those US/2014 strains were not identical, the other 28 polymorphisms identified in this study might still be associated with the severity of EV-D68. Moreover, 29 nonconserved residues were identified from TW/2014 strains, as shown in Fig. 3. Among them, positions 558, 868, 1031, 1141, and 1190 in 3 Linkou CGMH cases showed residues “G,” “A,” “S,” “T,” and “F,” respectively, which diverged from the other 8 TW strains, which contained residues “A,” “V,” “N,” “N,” and “Y.” This result demonstrated that the 3 EV-D68 cases (including case 11 with severe clinical symptoms) from Linkou CGMH had different genetic features from the other 8 Centers for Disease Control and Prevention, Taiwan, cases.

Rearrangements in the spacer region of the 5′ UTR in EV-D68 between the early 1960s and mid-1990s resulted in 2 deletions of 24 and 11 nucleotides.^[5]^ One Taiwanese strain in this study has a deletion at positions 721 to 728 (i.e., 8 nt long) that was shorter than the previously reported 1 that was 11 nt long. More studies are needed to investigate any association between this shorter deletion in the 5′ UTR and the efficiency of translation or virulence. We found that clade D strains had an N692 deletion and R860/L861 insertions in the *VP1* gene, including 5 China, 5 France, 1 Italy, 1 Japan, and 1 US strains. More complete genomes are needed to confirm these genetic features in the other clade D strains. In addition, the *VP1* gene also contains serotype-specific neutralization sites (e.g., BC and DE loops). No EV-D68/TW strains isolated in 2014 had any novel substitutions in these 2 loops; they were identical to most of the US/2014 outbreak strains.

To conclude, we performed phylogenetic and genetic diversity analyses on all available sequences to reveal the molecular phylogeny, diversity, and epidemiology of EV-D68, and contributed 11 new EV-D68 genomes for analysis. A new subclade, genetic indels, and polymorphisms were discovered, which further elucidate the molecular evolution of EV-D68. Although there was no direct evidence to prove virus variants causing clinical characteristics of patients infected by EV-D68, enterovirus studies indicated that virus variants may contribute to clinical manifestations, disease severity, and outbreaks, such as EV-71^[28,30]^ and coxsackievirus A6.^[35,36]^ In this decade, EV-D68 has been detected worldwide and has caused outbreaks and severe clinical symptoms. Further studies are needed to investigate the associations between virus variants and clinical outcomes in this globally reemerging virus.
